# Transplanted adipose-derived stem cells can be short-lived yet accelerate healing of acid-burn skin wounds: a multimodal imaging study

**DOI:** 10.1038/s41598-017-04484-0

**Published:** 2017-07-05

**Authors:** Ghulam Muhammad, Jiadi Xu, Jeff W. M. Bulte, Anna Jablonska, Piotr Walczak, Miroslaw Janowski

**Affiliations:** 1 Russell H. Morgan Department of Radiology and Radiological Science, Division of MR Research, the Johns Hopkins University School of Medicine, Baltimore, MD 21205 USA; 2 Cellular Imaging Section and Vascular Biology Program, Institute for Cell Engineering, the Johns Hopkins University School of Medicine, Baltimore, MD 21205 USA; 3 Stem Cell Laboratory, University of the Punjab, Lahore, Pakistan; 4 Zoology Department, Faculty of Sciences, Ghazi University, Dera Ghazi Khan, Pakistan; 5 F.M. Kirby Research Center, Kennedy Krieger Institute, Baltimore, MD 21205 USA; 6 Department of Biomedical Engineering, The Johns Hopkins University School of Medicine, Baltimore, MD 21205 USA; 7 Department of Chemical & Biomolecular Engineering, The Johns Hopkins University School of Medicine, Baltimore, MD 21205 USA; 8 Department of Oncology, The Johns Hopkins University School of Medicine, Baltimore, 21205 MD USA; 9 Department of Radiology Faculty of Medical Sciences, University of Warmia and Mazury, Olsztyn, Poland; 10 NeuroRepair Department, Mossakowski Medical Research Centre, Polish Academy ofSciences, Warsaw, 02-106 Poland

## Abstract

The incidence of accidental and intentional acid skin burns is rising. Current treatment strategies are mostly inadequate, leaving victims disfigured and without treatment options. Here, we have shown that transplantation of adipose-derived stem cells (ASCs) accelerates the process of acid burn wound-healing. Pre-conditioning of ASCs using ascorbic acid (AA) or hypoxic conditions provided additional benefit. While the wounds were ultimately healed in all mice, histological analysis revealed that, in non-transplanted animals, the number of hair follicles was reduced. Bioluminescent imaging (BLI) of transplanted ASCs revealed a gradual loss of transplanted cells, with a similar rate of cell death for each treatment group. The signal of fluorinated cells detected by a clinically applicable ^19^F MRI method correlated with the BLI findings, which points to ^19^F MRI as a reliable method with which to track ASCs after transplantation to skin wounds. No difference in therapeutic effect or cell survival was observed between labeled and non-labeled cells. We conclude that, despite being short-lived, transplanted ASCs can accelerate wound-healing and reduce hair loss in acid-burn skin injury. The fluorine nanoemulsion is a clinically applicable cell label capable of reporting on the survival of transplanted cells.

## Introduction

The incidence of acute burn injuries caused by fire, heat, electricity, and chemicals is increasing. The American Burn Association has reported that more than 450,000 victims of burn injuries receive medical treatment annually. Approximately 40,000 victims of acute burn injuries are hospitalized every year, and, among them, 4% of patients ultimately die. According to the World Health Organization report N 365, globally, more than 11 million people, including 100,000 to 500,000 victims of chemical injuries, require hospitalization, with 30% eventually facing death^1, 2^.

Cases of chemical burns are more prevalent in third-world countries, with women the predominant victims due to increasing violence for the sake of revenge or rivalry^3^. Due to their socially sensitive nature, the majority of the cases are not even reported. Typically, acids are thrown on the faces of females, resulting in permanent disfigurement, which leads to their social isolation and rejection from society. Treatment is highly ineffective and is based on early excision followed by the temporary application of a hydrocolloid dressing^4^. Due to very limited treatment options for these devastating chemical assaults, victims often commit suicide^5^.

There is growing interest in the use of stem cell-based regenerative medicine for wound-healing of skin injuries. Multiple studies have shown that mesenchymal stem cells (MSCs)^6^ from various sources are excellent candidates for the repair of damage to connective tissue^7–10^. In combination with various scaffolding materials, MSCs were shown to promote healing of various skin injuries^11–14^. For example, hypertrophic scarring was reduced following treatment with MSCs, and this effect was shown to be p53-dependent^15^.

While the use of an extracellular matrix in addition to MSCs may be beneficial, their use adds complexity and cost to treatment, preventing its wide utility in third-world countries.

In this study, we tested the efficacy of local injections of adipose-derived stem cells (ASCs) to the injured areas to facilitate the wound-healing process. Importantly, the ASCs fulfill the International Society for Cellular Therapy (ISCT) criteria for MSCs; however, they are distinct from bone marrow-derived MSCs^16^. The selection of ASCs was based on their better performance *in vitro* compared to bone marrow-derived MSCs in our previous study^17^. There is also a vast body of literature supporting the effectiveness of ASCs in the treatment of pressure ulcers^18, 19^, thermal burns^20–22^, and full-thickness skin wounds^23, 24^. In addition, it has been previously shown that pre-conditioning of ASCs with ascorbic acid (AA)^25, 26^ and hypoxia^27, 28^ increases the production of various growth factors; thus, we have added such experimental conditions to our study design.

The relatively superficial site of cell transplantation in skin wounds introduces the risk of leakage and loss of cells. Therefore, verifying proper placement and the persistence of cells over time is highly desirable for proper interpretation of any observed therapeutic effects. This information may be applied to customize therapeutic protocols, including reinjection in case of misinjection or the application of a booster dose.

There are various approaches to non-invasive cellular imaging^27^. There is usually needed a certain trade-off between sensitivity and specificity to obtain thoughtful images of transplanted cells. The magnetic resonance imaging (MRI) is characterized by the outstanding spatial resolution, so it facilitates the understanding the location of transplantation site and is very versatile as depending on the technique can be very sensitive or very specific. There is also no radiation incurred during obtaining MR images, what is especially valuable if repetitive imaging sessions are needed such as in case of stem cell tracking. Here, we compare a very sensitive but less specific superparamagnetic iron oxide (SPIO) with a very specific but less sensitive fluorine to learn about their usefulness for tracking of stem cells transplanted in a very specific conditions of the skin wound imaging. The concept of magnetic resonance imaging (MRI) of SPIO-labeled cells was introduced over two decades ago^28^ and has been especially useful for cell-tracking in the brain^29^ and lymph nodes^30–32^. However, the cell detection through image hypointensities within superficial areas of tissue injury can be complicated by the presence of susceptibility artifacts at air-tissue interfaces, such as the skin. This limitation may be overcome by the use of fluorine (^19^F) MRI, which can be characterized as a “hot spot” imaging technique without background signal^33, 34^. Here, the generally low sensitivity of ^19^F imaging can be improved by the close proximity of the RF coil and superficially located the transplanted cells, what can be a challenge for a deep transplantation sites such as brain or spinal cord. The recent clinical use of a ^19^F formulation has fueled further interest in broader applications^35^. We have previously performed extensive *in vitro* toxicity studies, which showed a lack of detrimental effects for mouse MSCs when labeled with SPIO nanoparticles or ^19^F nanoemulsions^17^. In this study, we tested the applicability of ^1^H and ^19^F MRI to track MSCs transplanted to acid-burn wounds. To this end, we used bioluminescent imaging (BLI) of luciferase-transfected MSCs as the gold standard for longitudinal assessment of cell survival in small animals^36, 37^, and correlated the BLI findings for each time point with clinically applicable MRI.

Rodent models of skin wounds are frequently used in experimental studies. However, skin retraction significantly contributes to wound-healing in rodents, in contrast to humans in whom re-epithelialization plays a major role. Thus, a method has been developed to limit the skin retraction and promote re-epithelialization in rodents through wound-splinting to better mimic the clinical scenario^38^. However, since the therapeutic effect of stem cells in this model has already been shown, and, in the current study, we focused more on stem cell imaging, we relied on the less complex model of skin incision, with no installment of additional devices that could potentially interfere with imaging.

## Materials and Methods

### Cell Preparation and Labeling

**Figure 1:**
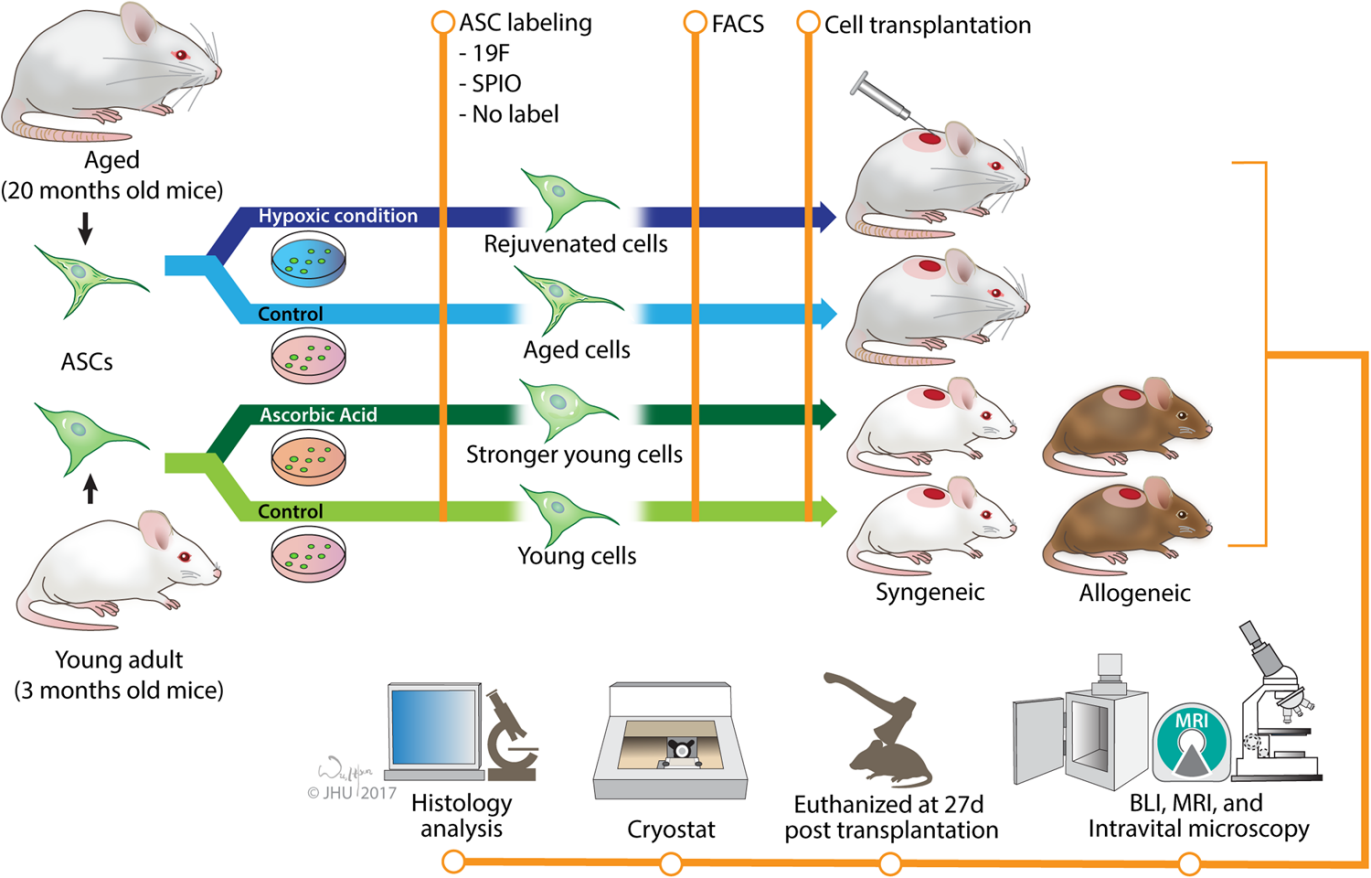
Study design.

This study was approved by the Institutional Animal Care and Use Committee at the Johns Hopkins University. The study design is illustrated in Fig. 1. ASCs were obtained from abdominal adipose tissue of three-month-old (young donor) and 20-month-old (adult donor) FVB luciferase-positive mice, as described previously^39^. Approximately 1–2 g of adipose tissue was first manually minced and subjected to digestion with collagenase-I solution for 1 h at 37 °C. Isolated ASCs were seeded in 25 cm^2^ flasks with Dulbecco’s Modified Eagle medium–low glucose (DMEM-LG) (Sigma, USA) supplemented with 15% fetal bovine serum (FBS) (HyClone, USA), 100 U/ml penicillin (Sigma, USA) and 100 µg/ml streptomycin (Sigma, USA) at a cell density of 1 × 10^5^ cells/ml and incubated at 37 °C and 5% CO_2_.

For AA preconditioning, ASCs were incubated with 250 µM L-ascorbic acid 2-phosphate sesquimagnesium salt hydrate (Sigma, USA) in complete culture media from passages 1 to 3, as reported previously^40^. For hypoxic preconditioning, ASCs were maintained in an incubator with oxygen concentration control (Thermo SCIENTIFIC, USA) at 3% oxygen from day 0 to passage 3, as described previously^41^.

For intravital multiphoton laser microscopy, cells were washed twice with PBS, trypsinized, washed and resuspended in 0.5 ml PBS (Gibco, USA) containing 1 µL Cell Tracker Green CMFDA (Life technologies, USA) for 30 minutes at 37 °C. Cells were washed twice with PBS and the final pellet was resuspended in PBS at the desired cell density.

For SPIO labeling, monolayers of ASCs grown in 25 cm^2^ flasks were washed twice with PBS (Gibco, USA) and incubated with complete growth medium supplemented with SPIO (MIRB, BioPAL, Inc, USA) at a concentration of 20 µg Fe/ml. After 24 hours, the medium was discarded and cells were washed twice with 1x PBS^42^. For fluorine-labeling, cells were incubated with growth medium without FBS containing 200 μl of Cell Sense ATM DM-Green (Celsense, USA) to 2 ml. After 24 hours, medium was discarded and cells were washed twice with 1 mM PBS^43^.

### Cell Characterization

Both SPIO- and ^19^F-labeled ASCs from the young animal group (see below), preconditioned with AA, were characterized for MSC markers by flow cytometry, as reported previously^44^. Briefly, ASCs at the third passage were trypsinized, washed with PBS, and incubated with primary antibodies for CD29, CD90, CD45, and CD34 (Biolegend, USA) for 15 min at room temperature. Isotype-specific antibodies were included as negative controls. Data were analyzed with Cell Quest Prodata analysis software (BD Biosciences, USA). At least three separate culture experiments run in duplicate were used for the analysis.

### Mouse Model of Acute Skin Acid-Burn Injury

Injury was induced in three-month-old (“young group,” n = 30) and 20-month-old (“adult group,” n = 16) mice. The young group consisted of either FVB (n = 15) or BALB/c (n = 15) male mice and the adult group consisted of an FVB group of male mice. Acute skin injury was induced as reported previously, with modifications^45^. Mice were anesthetized with isoflurane and their backs were shaved. A sterile filter paper of 0.5 cm^2^ was soaked in 12.06 N HCl (Merck) for one minute and applied at the dorsolateral side of the neck for one minute. Two wounds were induced on both sides of each animal. MSCs were unilaterally transplanted, with the other site serving as a control. Injured animals were kept in separate cages with access to food and water *ad libitum*.

### Cell Transplantation

Twenty four hours after injury, necrotic tissue was removed by scissor dissection under isoflurane anesthesia. These excisional wounds were approximately 0.5 cm^2^ in size. Animals were randomly divided into six groups: four young and two adult animal groups (see graphic abstract). The groups of adult animals included naïve and hypoxia pre-conditioned ASCs. The groups of young animals included a group of naïve and AA-preconditioned syngeneic grafts, and naïve and AA-preconditioned allogeneic grafts. Then, each of six groups of animals was further randomly selected to receive SPIO-labeled, ^19^F-labeled, or non-labeled ASCs. ASCs (1 × 10^6^ cells suspended in 50 µl saline) were transplanted intra-dermally in the border of the wound. A sham injection was given by injecting 50 µl saline to the contralateral side. Wounds were then dressed continuously with 3 M Tegaderm transparent film dressing until the wound disappeared (St. Paul, USA). The dressing was not changed during study duration to avoid inadvertent removal of transplanted ASCs during manipulation. All animals were euthanized for histological analysis on day 27, as reported previously^46^.

### Multi-Photon Imaging

Intravital imaging was performed using an Olympus FV1000MPE multiphoton laser-scanning microscope. Animals (n = 2) anesthetized with isoflurane in oxygen-enriched air were placed on a custom-made holder. A fragment of skin with transplanted cells was immobilized in a holder window, eliminating any respiratory animal movement. Ten minutes before imaging, TexasRed-conjugated dextran (Mw = 70 kDa, Life Technologies) was injected into the tail vein to visualize blood vessels. Animals were imaged every 10 minutes for two hours. For analysis of cell motility, individual CMFDA-labeled cells were randomly selected and the traveled distance between 0 and 120 minutes of observation was measured.

### Magnetic Resonance Imaging

Mice were imaged using a horizontal bore 11.7 T scanner (Bruker, Billerica, MA) equipped with actively shielded gradients with a maximum strength of 74 Gauss/cm. A 72 mm quadrature volume resonator (Bruker, Ettlingen, Germany) was used as a transmitter, and a 10 mm planar surface coil (Bruker, Germany) was used as a receiver^47^. All animals were anesthetized using 2% isoflurane for induction and 1–1.5% during scanning. Mice were placed on a water-heated animal bed equipped with temperature and respiratory control and their heads were immobilized using a custom holder with a bite bar and ear pins. T_2_*-weighted axial images were acquired using a FLASH sequence with the following parameters: field of view (FOV) = 2.2 × 2.2 cm; slice thickness = 1 mm; TR (repetition time)/TE (echo time) = 300/2.9 ms; number of averages (NA) = 2; and matrix size = 128 × 128.

^19^F MRI was performed using the same MRI scanner with a 10 mm double-tuned ^19^F-^1^H transceiver surface coil (Bruker, Germany). ^19^F images were obtained using a RARE (rapid acquisition with relaxation enhancement) sequence with TR/TE = 1000/4.5 ms; slice thickness = 2 mm; a matrix size = 32 × 32; FOV = 2.0 × 2.5 cm; and NA = 512. ^1^H T_2w_ MR images were acquired with a RARE sequence using the same geometry as ^19^F MRI and the following parameters: TR/TE = 5000/9 ms, and NA = 1. ^19^F MR images were overlaid on the anatomical T_2w_ images, and the area of positive hot-spot ^19^F MRI signal was calculated using Image J software by selecting pixels with signal intensity above the background.

### Bioluminescent Imaging

Bioluminescent imaging was conducted as previously reported^37^. Briefly, BLI was conducted every 48 hours until day 22 after stem cell transplantation using an IVIS Spectrum/CT scanner (Perkin Elmer). Luciferin was administered intraperitoneally at 150 mg/kg and images were acquired every five minutes for up to 30 minutes to reach the signal peak. The exposure time was set at one minute, with the data represented as photon flux (photons/s).

### Therapeutic Evaluation

Wound reduction analysis was performed as reported previously^48, 49^. Briefly, starting at day 5 post injury, wound boundaries at different days were traced on glass slides and copied on millimeter-indexed graph paper. The calculated percentage of wound closure was determined from the initial and final drawn areas. The rate of wound-healing was calculated as the percentage of wound closure = (area of initial wound − area of final wound/area of initial wound) × 100. The period to complete wound closure was measured from the initial injury to the complete wound closure, as described previously^50^.

### Post-Mortem Analysis

H&E staining was performed to analyze the formation of granulation tissue and the skin-healing process with respect to the development of basic skin components. On day 27 post-transplantation, animals were transcardially perfused, skin samples were dissected and fixed in 4% paraformaldehyde (Sigma, USA) overnight at 4 °C, and then transferred to 30% sucrose (Fisher Scientific, USA) for 48 hours at 4 °C. Then, samples were frozen in crushed dry ice and kept in the freezer at −70 °C until embedded in an optimal cutting temperature compound and cryo-cut at 20µm-thick slices. For histological scoring, we focused on hair follicles, as these, along with the sebaceous glands, are responsible for the basic skin functions of thermoregulation and immunomodulation. At least four images per wound were taken randomly and the number of hair follicles was counted for each image^51, 52^.

For detection of transplanted luciferase-positive cells, cryosections were washed thrice with PBS for five minutes each followed by two hours blocking at room temperature with 3% bovine serum albumin (Sigma, USA) and 0.1% triton at a 1:10 concentration. Rabbit polyclonal anti-luciferase primary antibody (211761, Abcam, USA), diluted 1:1500 in blocking solution, and was incubated overnight at room temperature followed by washing thrice with PBS for five minutes each. Anti-rabbit secondary antibody, diluted 1:1000 in blocking solution, was incubated for two hours at room temperature. Slides were treated with 500 μl DAPI per slide for 10 minutes at room temperature followed by washing. Coverslips were mounted with fluorogel (Electron Microscopy Sciences, USA) as an embedding medium. Thirty fluorescent images per group were acquired to quantify transplanted cells.

### Statistical Analysis

PROC MIXED (SAS) was used for statistical analysis, with the lowest means square (LMS) test for comparison between groups. The statements “repeated” and “random” were used for repeated measures and to express random effects, respectively. PROC CORR (SAS) was employed for correlation analysis using the r-Pearson correlation coefficient. Logarithmic transformation of BLI data was performed to improve data normalcy.

## Results

### Characterization Of ASCs

The ASCs were characterized by the high level of expression of MSC-specific markers: CD90 and CD29, and the minimal expression of HSC-specific markers: CD34 and CD45. Preconditioning of ASCs with AA and labeling of non-preconditioned and AA-preconditioned ASCs did not affect the expression of the above-mentioned markers (Suppl. Table 1).

### Therapeutic Effects

#### Time to wound closure

**Figure 2:**
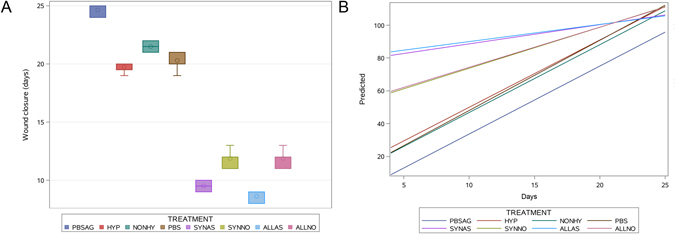
The wound healing: time to wound closure presented as a box plots (**A**), and regression analysis of wound reduction with predicted values of various treatment groups plotted versus time, expressed as days for various treatment options (**B**). SYNAS – young, syngeneic cells + AA, ALLAS – young, allogeneic cells + AA, SYNNO – young, syngeneic, non-preconditioned cells, ALLNO – young, allogeneic, non-preconditioned cells, PBS – control side young animals, HYP – adult, hypoxic, NONHY – adult, non-hypoxic, PBSAG – control side in adult animals. The boxes represent interval between 25 and 75 percentile, and whiskers the minimum and maximum values.

The time to complete wound closure in adult mice equaled 24.6 ± 0.5 days, and transplantation of syngeneic ASCs derived from adult mice decreased that time to 21.5 ± 0.5 days. Hypoxic preconditioning of ASC further accelerated healing to 19.8 ± 0.5 days. The difference between groups was statistically significant (p < 0.05) (Fig. 2A).

The time to complete wound closure in young mice was 20.3 ± 0.6 days, and transplantation of ASCs significantly decreased the healing time to 10.4 ± 1.6 days (p < 0.05). Interestingly, multivariate regression showed that the relation of the donor to the recipient did not influence time to complete wound closure: values for allogeneic vs syngeneic were 10.1 ± 1.77 and 10.6 ± 1.35 days (p = NS), respectively, whereas preconditioning by AA decreased the healing period from 11.9 ± 0.67 to 9.1 ± 0.68 days (p < 0.05) (Fig. 2A). Cell labeling did not have any impact on the time to complete wound closure in either adult or young animals (age = random variable; label = fixed variable), with p = 0.35.

#### Wound reduction

**Figure 3:**
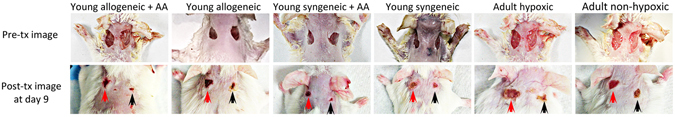
Representative images of the wound-healing process at day 9 across all experimental groups.

While the time to complete wound closure allows for a definite estimation of treatment efficacy, does not provide any information about the dynamics. Therefore, the daily-examined wound reduction profile allowed for a more detailed insight into the healing process. The analysis of all treatment groups revealed significant effects both for treatment type and the time post-transplantation (Fig. 2B). At day 9, there were clear differences between wound sizes (Fig. 3). Regression analysis indicated that the most effective treatment was a combination of young cells preconditioned in ascorbic acid, transplanted to a young recipient (regardless of whether syngeneic or allogeneic). The position of the lines on Fig. 2B indicates that these animals gained the most therapeutic effect very early on (before wound measurements were introduced at day 5). The young non-preconditioned cells, regardless of being syngeneic or allogeneic, grafted to a young recipient, resulted in a slightly, but statistically significantly delayed therapeutic effect. In adult animals treated with adult animal-derived ASCs, for both non-preconditioned and preconditioned with hypoxia, the course of healing was similar to that in young control animals (PBS administration). The lowest scores for control adult animals (PBS administration) indicated that the animals gained a therapeutic effect very slowly at the beginning. Overall, the regression lines suggest that transplanted cells were especially useful for very early promotion of wound-healing, while, at later time points, non-transplanted animals were also capable of effective wound-healing, in our model.

**Figure 4:**
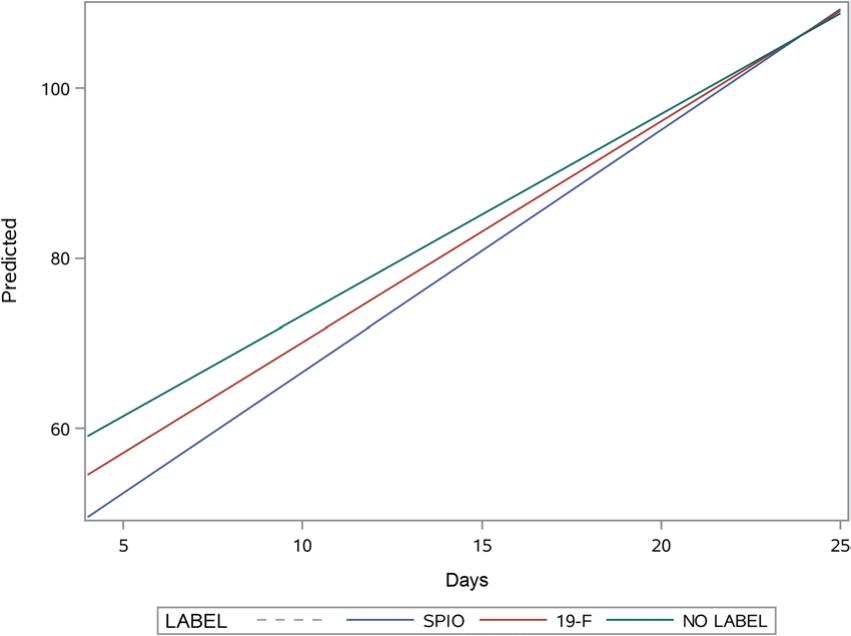
Regression analysis with predicted values of therapeutic effect (wound closure) for the different imaging groups versus time, expressed as days.

While cell labeling did not have any impact on the final therapeutic effect (see above), there were observed differences in the time course, with non-labeled cells having an earlier time course of therapeutic effects compared to SPIO-labeled cells (p = 0.008). The therapeutic time course of ^19^F-labeled cells was somewhere in-between, with no statistical significance compared to non-labeled and SPIO-labeled cells (Fig. 4).

#### Histopathological analysis

**Figure 5:**
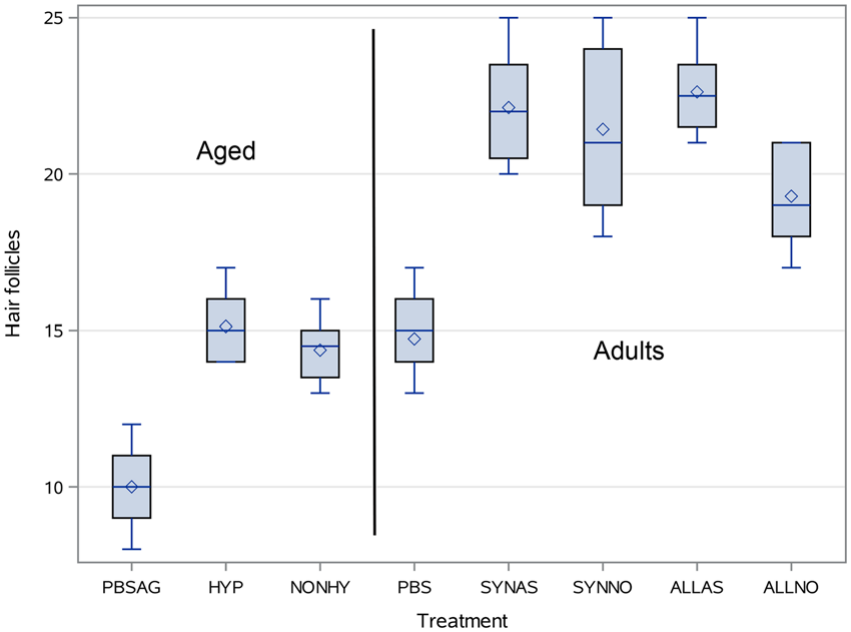
The number of hair follicles in healed wounds. SYNAS – young, syngeneic cells + AA, ALLAS – young, allogeneic cells + AA, SYNNO – young, syngeneic, non-preconditioned cells, ALLNO – young, allogeneic, non-preconditioned cells, PBS – control side young animals, HYP – adult, hypoxic, NONHY – adult, non-hypoxic, PBSAG – control side in adult animals. The boxes represent interval between 25 and 75 percentile, and whiskers the minimum and maximum values.

While visual inspection of the surface of all wounds revealed effective healing, with newly formed skin indistinguishable from the intact skin areas, the histological analysis (Suppl. Fig. 1) with quantification of hair follicles detected differences between the animal groups (Fig. 5). The lowest number of hair follicles was observed in the control adult animals (10 ± 1.41), which significantly increased (p < 0.05) in adult animals transplanted with non-preconditioned adult cells (14.37 ± 1.06) and adult cells preconditioned by hypoxia (15.12 ± 1.12), without a significant difference between the two transplanted groups (p = NS). The young animals transplanted with young AA-preconditioned cells revealed the highest number of hair follicles (22.37 ± 1.59), while non-preconditioned cells were statistically less efficient (20.35 ± 2.34) (p < 0.05). Non-transplanted young animals recovered to the level of treated adult animals (14.4 ± 0.98). As in the case of wound closure, there was also no difference between the transplantation of syngeneic and allogeneic cells (21.8 ± 2.18 vs 21.06 ± 2.22). No differences between non-labeled, SPIO-, and ^19^F-labeled cells in adult and young populations were observed (Suppl. Tab. 2, p = NS). Immunohistochemistry confirmed the lack of transplanted cells in the tissue sections (data not shown).

### Cellular Imaging

#### Intravital multiphoton microscopy of transplanted cells for localization and migration of ASCs

The effective intradermal placement of ASCs was confirmed by multiphoton microscopy in live mice with no spontaneous leakage over two hours post-transplantation. Actually, no cell migration was observed during that time (Suppl. Fig. 2). The mean distance between selected cells was 385 ± 223 µm, and, over two hours of observation, this changed only 13.22 ± 27.18, which was not statistically significant (p = NS).

#### The measurement of survival of intra-dermally transplanted ASC by BLI

**Figure 6:**
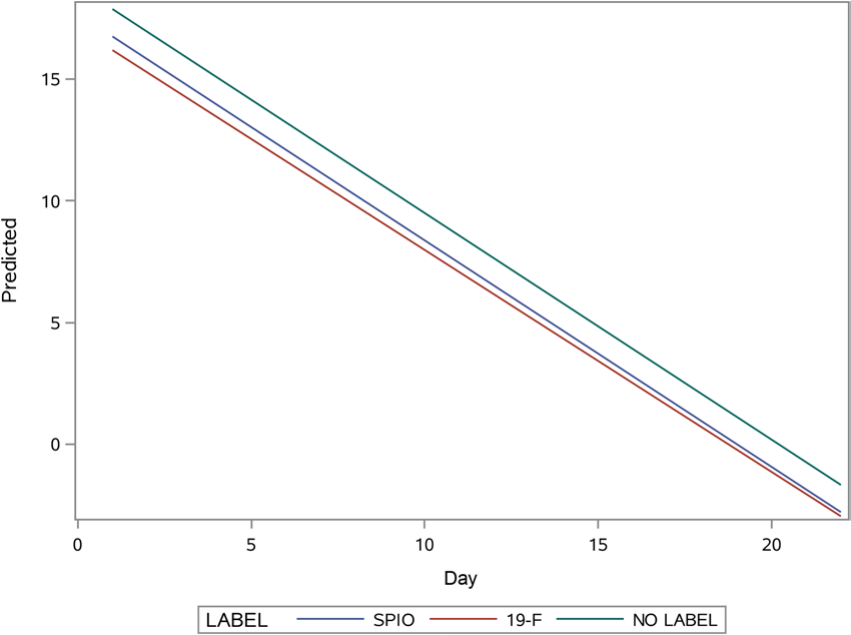
Regression analysis with predicted values of cell death for the imaging methods plotted versus time, expressed as days.

For the analysis of cell death, the time course of the BLI signal was analyzed. There was no observed interaction between time or cell labeling method (p = 0.95), (Fig. 6), nor between time and age (p = 0.19), which indicates that the dynamics of the drop in the signal were nearly identical in all groups (Fig. 6). As also expected, there was no influence of cell preconditioning (p = 0.35), or donor-recipient relation (p = 0.1). Thus, the speed of the death of transplanted cells is quite universal and is not affected by any of the investigated factors.

#### The value of MRI for assessment of location and survival of intra-dermally transplanted ASCs

**Figure 7:**
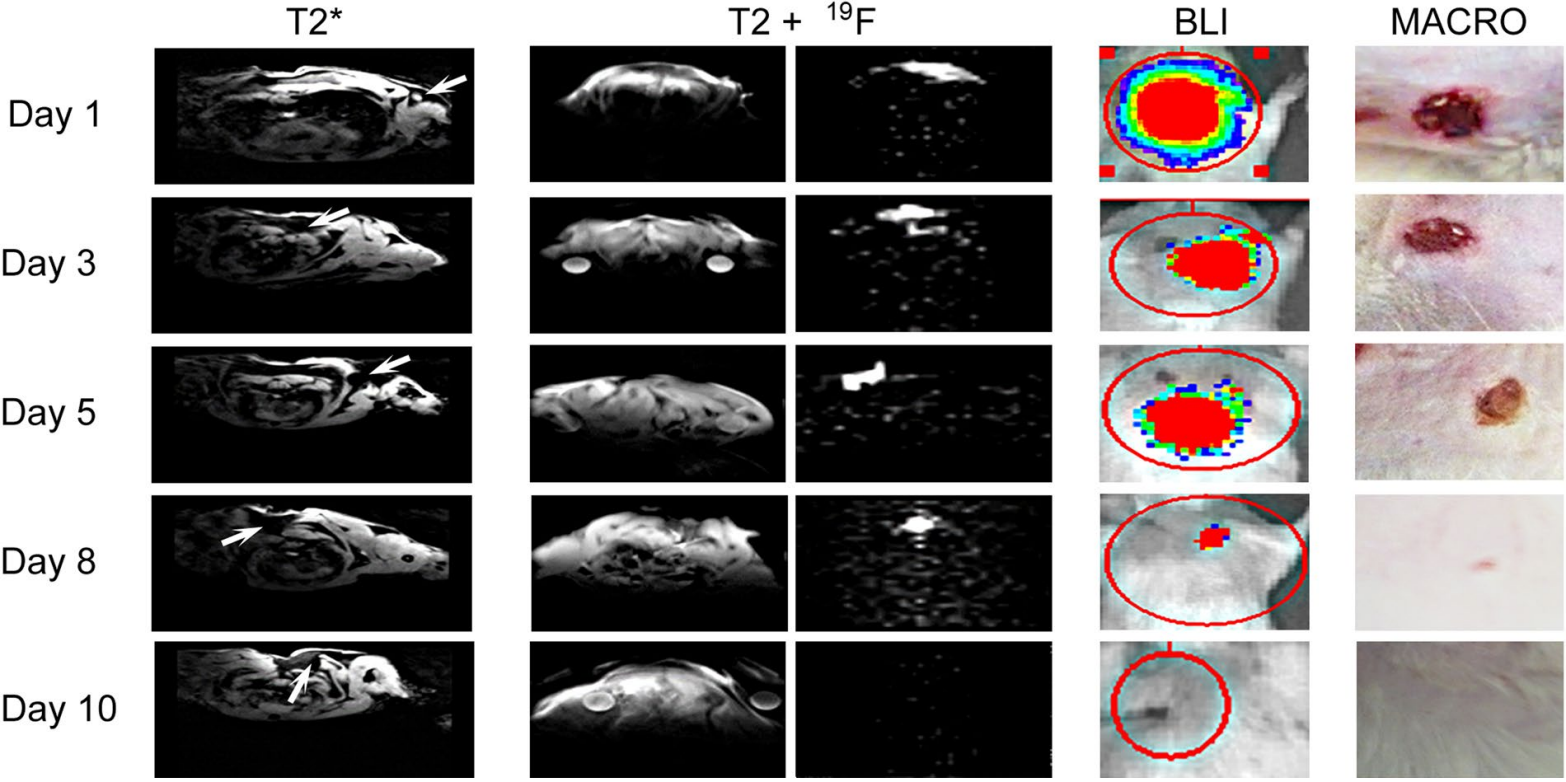
Imaging time course of ASCs and macrophotographs showing the process of wound-healing on an example of transplantation of AA-preconditioned, syngeneic, SPIO- or ^19^F-labeled ASCs to young animals. The ^19^F images, BLI, and MACRO pictures belong to the same animal, while T2* images were derived from another animal. Arrows indicate the hypothetical location of iron oxide-labeled cells on T2* MRI, but data are inconclusive. ^19^F MRI shows clear hot spot images of cell deposits. T2w MRI presents an anatomical reference for ^19^F MRI. The BLI signal is represented as a color-coded map. The macro pictures depict the time course of wound-healing.

**Figure 8:**
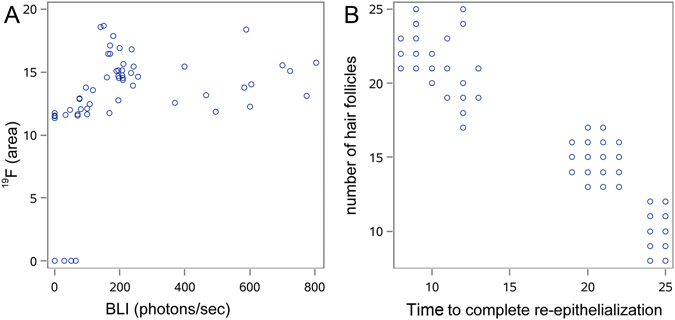
Correlational analyses. Correlation of BLI and 19 F signal loss (**A**) and correlation of *in vivo* and post mortem outcome measures (**B**).

Representative images of the time course of ^1^H MRI and ^19^F MRI cellular MRI, BLI, and wound reduction are shown in Fig. 7. Susceptibility artifacts, due to the micro-hemorrhages present in injured tissue, deteriorated the quality of ^1^H MR images to a level that was not interpretable and transplanted cells were difficult to separate from the background at any of the investigated time points. In contrast, ^19^F MRI was capable of a robust detection of transplanted cells within the tissue, as represented by background-free, hot-spot signals (Fig. 7). We found a good correlation between the course of the disappearance of the BLI and ^19^F MRI signals (r = 0.6, p < 0.05) (Fig. 8A). ^19^F MRI, which is a clinically applicable imaging modality, may thus provide valid information about the successful placement and survival of intra-dermally transplanted ASCs.

### Correlation Between Cell Survival and Therapeutic Effect

The length of cell survival negatively correlated with the *in vivo* therapeutic outcome, including the time to complete wound closure (r = −0.45, p < 0.001) and the number of hair follicles (r = −0.47, p < 0.001). This paradox indicates that a shorter life-span of transplanted cells is more beneficial. Both *in vivo* imaging and histology outcome measures were well correlated (Fig. 8B).

## Discussion

We have shown here that the transplantation of stem cells is beneficial for the healing of acid-burn skin wounds. Assessment of cell viability with BLI demonstrated that shorter survival times for transplanted cells were not related to a decrease in therapeutic efficacy. We also confirmed that labeling of ASC with either SPIO nanoparticles or ^19^F nanoemulsions did not alter therapeutic outcome. Labeling with SPIO nanoparticles is impractical for proper monitoring of the presence and persistence of cells, due to the difficulty in distinguishing between the endogenous hypointensities related to microhemorrhagic injury and the hypointense transplanted cells. In contrast, ^19^F MRI was able to report not only the location of transplanted cells, but also their survival. The source of cells, i.e., allogeneic or syngeneic, did not affect the length of cell survival nor the therapeutic activity of transplanted ASCs, indicating that the cause of cell death was not related to an adaptive immune response.

Surprisingly, in animals in which transplanted cells died more rapidly, the therapeutic effect was greater. This might be related to the slight difference in the status of the innate immune system of animals, wherein some with a stronger innate immune reaction that eliminated cells more effectively, also healed better. However, this might also depend on the transplanted cells themselves, as more stressed cells die faster, immediately releasing beneficial soluble factors facilitating tissue healing. The latter hypothesis is consistent with the current consensus that healing may not be a result of cell replacement, but rather from paracrine effects. There are recent reports describing therapeutic effects of transplanted cells despite their short survival^53–55^.

The lack of difference in the survival of syngeneic vs. allogeneic stem cells seems counterintuitive; however, their short survival of up to 10 days is too short for the development of an adaptive immune response, which requires about 14 days^36^. The equal effectiveness of allogeneic and syngeneic ASCs is attractive from a clinical point of view, as off-the-shelf vials of ASCs could be used for therapy for acid-burned skin, a serious injury where there is no time to wait for expansion of autologous ASCs. Intradermal transplantation of stem cells is a methodologically simple procedure. However, the relatively superficial location of the intradermal space requires an imaging method to confirm that transplanted cells remain in place and do not leak out during or after the injection procedure. The presence of transplanted cells in tissue can be readily assessed in small-animal models by BLI, but this method is not clinically translatable or applicable in large animals due to the limitations of light penetration in deeper tissues. We found that intravital microscopy was also able to detect CMFDA-labeled cells, but the limited imaging depth does not allow any insight into the cells during the ongoing process of wound closure. Hence, we were interested to find a way to determine the persistence and survival of ASCs with a clinically applicable methodology.

The preconditioning of ASCs did provide additional therapeutic effect, but this additional effect was relatively small, compared to the effect of using ASCs themselves, especially in a young animal setting, so we would not recommend using preconditioning for initial clinical studies. The search for better preconditioning protocols could be also considered.

MRI is a widely used imaging modality with which to track SPIO-labeled cells, but this approach is not able to distinguish between dead or live cells^56, 57^, although it is capable of detecting rejection of pancreatic islets deposited under the renal capsule in a primate model of diabetes^58^. However, the previous studies were based on transplantation of SPIO-labeled cells in relatively homogeneous, non-injured tissues, and we have shown here that SPIO-labeling is not useful for transplantation to acutely injured tissues bearing microhemorrhages. “Hot-spot” ^19^F MRI has been recently shown to be capable of detecting transplanted immune cells in a clinical setting^35^. A recent *in vitro* study revealed that ^19^F MRI may be capable of reporting on cell survival, with the fluorine signal dissipating upon both induced apoptosis and necrosis^59^. However, others have shown that the ^19^F signal may persist in the brain after the death of transplanted cells^60^. We have shown that ^19^F MRI was able to overcome the difficulties of ^1^H MRI detection of SPIO-labeled cells and was able to precisely locate ^19^F-labeled cells. Moreover, in our setting, ^19^F MRI was also capable of reporting on the survival of transplanted cells, as validated by BLI. Further studies are needed to determine whether there is a difference in the clearance of fluorinated nanoemulsions between the brain and other parts of the body. It is possible that the blood-brain barrier or the lack of lymphatic vessels do not allow for rapid clearing of the fluorine label following cell death in the brain.

We did not test whether ASCs derived from young donors are capable of adding additional benefit to adult recipients compared to ASCs derived from adult donors. However, we thought that the finding that allogeneic ASCs were as effective as syngeneic ASCs eliminates the need to make the effort to design therapies based on autologous ASCs. The ability to use an off-the-shelf allogeneic product and the fact that allogeneic cells are derived from young donors indicates that such an additional experimental group would not provide any clinically important information, so we abandoned it.

^19^F MRI is also capable of assessing quantitatively the amount of fluorine, which could also be advantageous in certain circumstances, but it requires a less sensitive volume coil to be used for imaging. However, since the sensitivity of ^19^F MRI is still an issue, we decided to use a more sensitive surface coil to detect ^19^F signal, but the dependence of the signal on the distance from the coil does not permit the calculation of the total number of fluorine atoms. It was a trade-off and we sacrificed the possibility for quantitative assessment of the fluorine signal to be able to see and delineate even fewer cells, thus enabling a more precise correlation with highly sensitive BLI. The area/volume of grafts has already been demonstrated as a useful parameter with which to follow the rejection process, so we feel that the provided data are of sufficient scientific quality^58^.

A limitation of our study is that, although we were able to test the beneficial effects of transplanted ASCs for improving the time course of wound-healing and the number of hair follicles, we could not test their ability to prevent the formation of scars. We showed that a beneficial effect can be achieved, even after a single-point injection, but the dose dependency of the cell concentration on wound repair and recovery will need to be addressed in the future. A larger acid-burn skin wound may present a different scenario, and large-animal studies are warranted to further assess the therapeutic value of ASCs for clinical translation.

## Conclusions

Intradermal transplantation of either syngeneic or allogeneic ASCs promotes wound healing in acid-burn skin injury. Pre-conditioning of ASCs with hypoxia or AA can provide an additional therapeutic effect. ^19^F MRI is well-suited for the monitoring of intradermally transplanted, fluorinated MSCs, while endogenous susceptibility effects in the wound area confound the interpretation of the hypointense signal from SPIO-labeled cells. Taking together, the intradermal transplantation of allogeneic, off-the-shelf, ^19^F-labeled ASCs is a compelling therapeutic opportunity, which could potentially be readily added to the current treatment protocols in order to accelerate healing of acid-burn skin injuries. However, further large animal studies could provide additional information about potential anti-scarring effects, thus better defining patients’ expectations.

### Declarations

#### Ethics approval and consent to participate

All animal procedures were approved by Johns Hopkins Institutional Animal Care and Use Committee in compliance with the Animal Welfare Act regulations and Public Health Service (PHS) Policy.

#### Availability of data and material

The datasets analyzed during the current study are available from the corresponding author on reasonable request.

## Electronic supplementary material

Supplementary information
