# Supplementary material for: Transplanted adipose-derived stem cells can be short-lived yet accelerate healing of acid-burn skin wounds: a multimodal imaging study
Source: Sci Rep. 2017 Jul 5;7:4644. doi: 10.1038/s41598-017-04484-0 (PMC5498606; doi:10.1038/s41598-017-04484-0)
Supplement: Supplementary file 1 — Supplementary information [file 41598_2017_4484_MOESM1_ESM.doc]

**Transplanted adipose-derived stem cells can be short-lived yet accelerate healing of acid-burn skin wounds: a multimodal imaging study**

Ghulam Muhammad^1,2,3^, Jiadi Xu^4^, Jeff W. M. Bulte^1,2,4,5-7^, Anna Jablonska^1,2^, Piotr Walczak^1,2,8^, Miroslaw Janowski^1,2,9^

*^1^Russell H. Morgan Department of Radiology and Radiological Science, Division of MR Research, the Johns Hopkins University School of Medicine, Baltimore, MD 21205, USA*

*^2^Cellular Imaging Section and Vascular Biology Program, Institute for Cell Engineering, the Johns Hopkins University School of Medicine, Baltimore, MD 21205, USA, ^3^Stem Cell Laboratory, University of the Punjab, Lahore, Pakistan, ^4^F.M. Kirby Research Center, Kennedy Krieger Institute, Baltimore, MD 21204, USA, ^5^Department of Biomedical Engineering, The Johns Hopkins University School of Medicine, Baltimore, MD 21205, USA^, 6^Department of Chemical & Biomolecular Engineering, The Johns Hopkins University School of Medicine, Baltimore, MD 21205, USA^, 7^Department of Oncology, The Johns Hopkins University School of Medicine, Baltimore, MD 21205, USA, ^8^Department of Radiology, Faculty of Medical Sciences, University of Warmia and Mazury, Olsztyn, Poland*

*^9^NeuroRepair Department, Mossakowski Medical Research Centre, Polish Academy of Sciences, Warsaw 02-106, Poland,*

**Corresponding author**

Miroslaw Janowski, MD/PhD

Assistant Professor

Russell H. Morgan Department of Radiology and Radiological Science

Division of MR Research

Institute for Cell Engineering

Johns Hopkins University School of Medicine

Miller Research Building Rm 649

733 N Broadway

Baltimore, MD 21205

Phone (443) 287 8461
Fax (443) 287 79 45
Email [mjanows1@jhmi.edu](mailto:mjanows1@jhmi.edu)

**Key Words:** mesenchymal stem cell, MRI, superparamagnetic iron oxide, fluorine, cell tracking, adipose tissue, acid burn, skin wound

**Running title:** Short-lived mesenchymal stem cells heal acid burns

Supplementary Table 1. ADMSC expression of MSC- (CD29 and CD90) and HSC (CD45 and CD34)-specific markers.

|  | CD29 (%) | CD90 (%) | CD45 (%) | CD34 (%) |
| --- | --- | --- | --- | --- |
| ADMSC | 94.6 | 98.2 | 0.7 | 4.9 |
| AA-ADMSC | 99.6 | 98.2 | 12.0 | 0.4 |
| ADMSC SPIO-labeled | 99.9 | 99.7 | 0.7 | 2.1 |
| AA-ADMSC SPIO-labeled | 98.9 | 99.9 | 8.5 | 2.3 |
| ADMSC ^19^F-labeled | 97.0 | 99.3 | 13.7 | 1.8 |
| AA-ADMSC ^19^F-labeled | 99.5 | 99.6 | 0.9 | 1.7 |

Supplementary Table 2. The number of hair follicles after treatment with unlabeled and SPIO- or ^19^F-labeled cells in adult and young mice.

|  | Non-labeled | SPIO-labeled | ^19^F-labeled |
| --- | --- | --- | --- |
| Adult | 15.33±1.03 | 14.33±1.03 | 14.5±1.29 |
| Young | 21.36±1.94 | 22.25±2.12 | 20.75±2.66 |

Supplementary figure captions:

**Suppl. Fig. 1.** Representative images of skin histopathology for the various treatments in the young animal groups. AA – ascorbic acid, ALLO – allogeneic, SYN – syngeneic, 19F – cells labeled with fluorine, SPIO – cells labeled with SPIO, NO LABEL – cell not labeled at all, SALINE – the wounds injected with saline, CONTROL – the skin sampled from the area of healthy skin. The arrows point on hair follicles. Note that there is some variability in the positioning of sections, so there is difference in the appearance of hair follicles among the images.

**Suppl. Fig. 2.** Intravital microscopy of intra-dermally transplanted cells at the times 0 (A) and 120 min (B) of the experiment (B) and respective images of dextran (C,D). Green – CMFDA-labeled ADMSC, Red: dextran-labeled vessels.


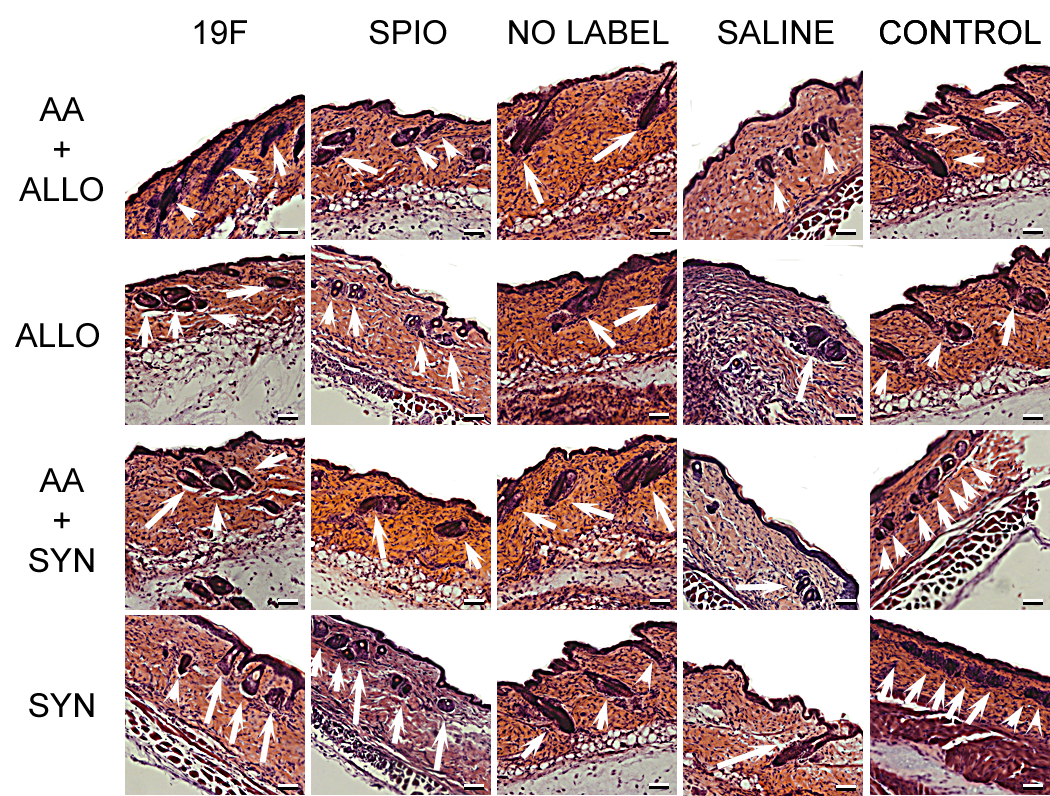


Figure 1


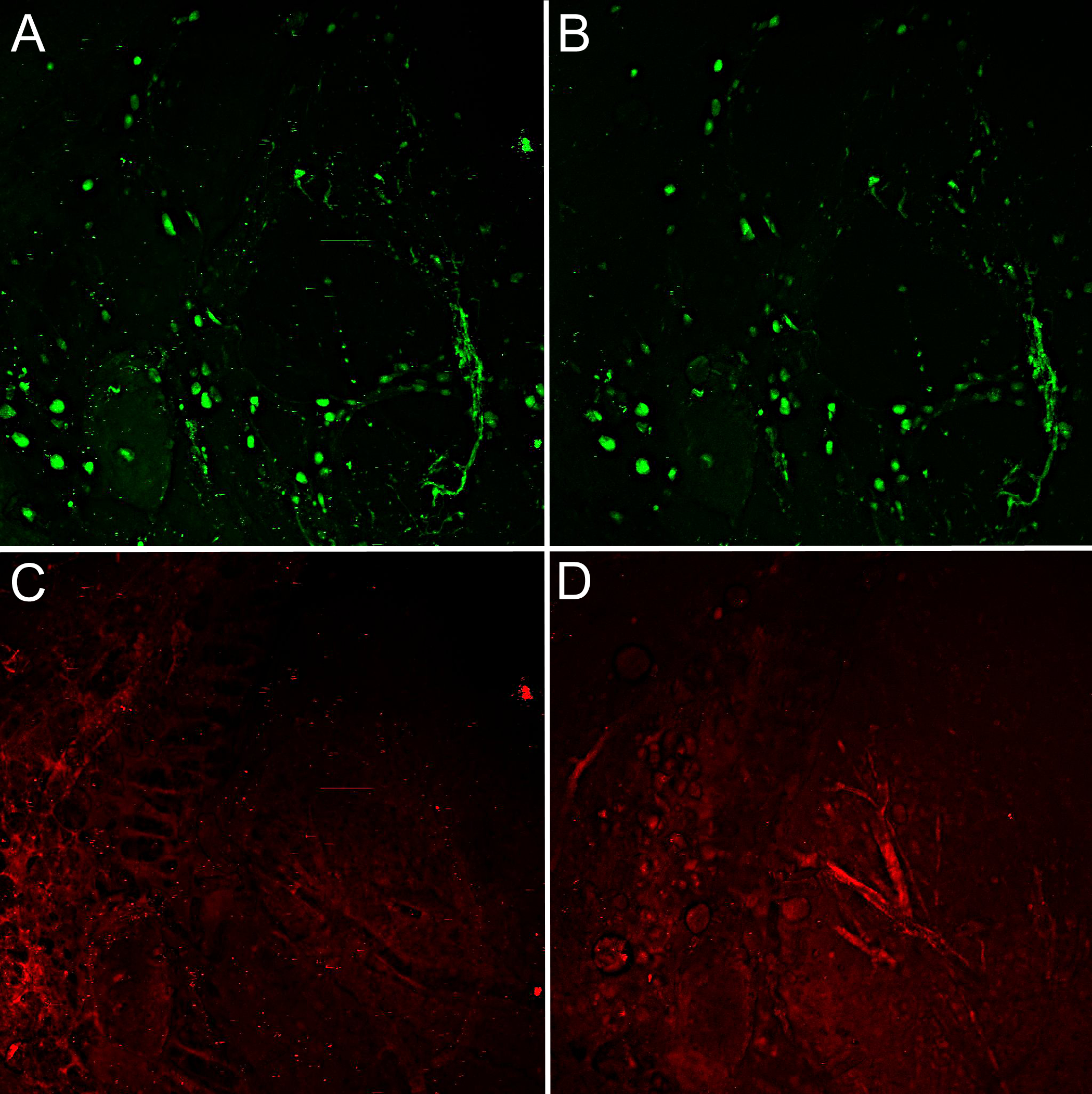


Figure 2
